# Cortical gyrification pattern of depression in Parkinson’s disease: a neuroimaging marker for disease severity?

**DOI:** 10.3389/fnagi.2023.1241516

**Published:** 2023-11-14

**Authors:** Qin Shen, Haiyan Liao, Sainan Cai, Qinru Liu, Min Wang, Chendie Song, Fan Zhou, Yujing Liu, Jiaying Yuan, Yuqing Tang, Xu Li, Jun Liu, Changlian Tan

**Affiliations:** Department of Radiology, The Second Xiangya Hospital, Central South University, Changsha, China

**Keywords:** Parkinson’s disease, depression, severity of depression, magnetic resonance imaging, cortical gyrification

## Abstract

**Background:**

Although the study of the neuroanatomical correlates of depression in Parkinson’s Disease (PD) is gaining increasing interest, up to now the cortical gyrification pattern of PD-related depression has not been reported. This study was conducted to investigate the local gyrification index (LGI) in PD patients with depression, and its associations with the severity of depression.

**Methods:**

LGI values, as measured using FreeSurfer software, were compared between 59 depressed PD (dPD), 27 non-depressed PD (ndPD) patients and 43 healthy controls. The values were also compared between ndPD and mild-depressed PD (mi-dPD), moderate-depressed PD (mo-dPD) and severe-depressed PD (se-dPD) patients as sub-group analyses. Furthermore, we evaluated the correlation between LGI values and depressive symptom scores within dPD group.

**Results:**

Compared to ndPD, the dPD patients exhibited decreased LGI in the left parietal, the right superior-frontal, posterior cingulate and paracentral regions, and the LGI values within these areas negatively correlated with the severity of depression. Specially, reduced gyrification was observed in mo-dPD and involving a larger region in se-dPD, but not in mi-dPD group.

**Conclusion:**

The present study demonstrated that cortical gyrification is decreased within specific brain regions among PD patients with versus without depression, and those changes were associated with the severity of depression. Our findings suggested that cortical gyrification might be a potential neuroimaging marker for the severity of depression in patients with PD.

## 1. Introduction

Depression is one of the most common non-motor symptoms of Parkinson’s disease (PD), which occurs in up to 40.4% for outpatients and 54.3% for inpatients with PD (Prange et al., 2022). It may be encountered at every stage of the disease and even precede the onset of motor symptoms (Thobois et al., 2017; Jeong et al., 2021). Beyond its negative influence on emotion, depression in PD is reported to be associated with higher motor-related disability and various complications such as dementia in the late stage of disease (Assogna et al., 2020). Nevertheless, the underlying pathophysiological relationship between PD and depression remains poorly understood.

Since the advent of magnetic resonance imaging (MRI), various approaches have been developed, making it possible to identify morphometric and functional changes non-invasively in the human brain *in vivo*. Previous functional MRI studies have suggested abnormal neural activity or connectivity in various brain areas which mainly involve the prefrontal-limbic circuit, the striatal-thalamic-prefrontal circuits and the basotemporal limbic circuits (Weintraub and Burn, 2011; Wen et al., 2016; Thobois et al., 2017).

As functional alteration tends to be influenced and constrained by the large-scale anatomical structure of the cortex (Honey et al., 2009), morphometric structural imaging studies may increase the neurobiological understanding of PD-related depression. The most commonly used methods for estimating the indices of cortical morphology such as voxel-based morphometry (VBM) and surface-based morphometry (SBM) have been used to identify brain morphometric changes in PD-related depression using three-dimension (3D) structural MRI. VBM is a typical method to estimate gray matter volume, as well as the density of brain structures (Ashburner and Friston, 2000), whereas SBM can provide more detailed information on brain morphometry including cortical thickness, area and gyrification (Dale et al., 1999; Fischl et al., 1999a,b).

Earlier studies using VBM explored the volume and density of cortical or subcortical structures in depressed PD (dPD) patients. Feldmann et al. (2008) detected density alterations of the bilateral orbitofrontal, bilateral rectal gyrus and the right superior temporal pole in dPD relative to non-depressed PD (ndPD) patients. Another VBM study reported that the severity of depression in PD patients negatively correlated with the volume of the limbic system structures, while positively correlated with the volume of the anterior cingulate (Van Mierlo et al., 2015). However, Kostić et al. (2010) found tissue loss in several white matter regions within the cortical–limbic network rather than in grey matter regions in dPD vs. ndPD patients. Only a few SBM studies, mainly focusing on cortical thickness, have assessed the cortical morphometry in dPD patients, and the reported results were controversial. For example, Chagas et al. (2017) and Luo et al. (2016) reported reduced cortical thickness in the left anterior cingulate gyrus and the left prefrontal area, respectively, in dPD relative to ndPD patients. On the contrary, Zanigni et al. (2017) found that compared with ndPD, the dPD patients had increased, rather than decreased cortical thickness within bilateral precuneus gyrus, and there was a positive correlation between cortical thickness in that region and the severity of depression. Additionally, according to the study of Huang et al. (2016), there was no significant difference in the whole-brain cortical thickness between patients with dPD and ndPD. These inconsistencies may due to several factors. First, human cortex is composed of multilayers of neurons, the cellular atrophy or death might be unequally through out all cortical layers under pathological conditions. Additionally, inter-species studies have demonstrated that cortical thickness tends to be more constant or with minimal changes relative to significant increase in cortical volume or folding with widely differing brain sizes (Im et al., 2008; Gautam et al., 2015). Thus, cortical thickness may be less sensitive in areas where cellular pathology is not transmural (Sterling et al., 2016). Furthermore, thickness measurement may not reflect more complex alteration of cortical morphological architecture (Van Essen, 1997; Gautam et al., 2015; Van Essen, 2020).

Gyrification, the process by which the morphological surface on brain undergoes changes to create sulcal and gyral regions (White et al., 2010), is believed to reflect the effective cortico-cortical connectivity, as well as optimal cortical-to-subcortical communications, with the minimal length of the connecting fibers (Klyachko and Stevens, 2003; Zhang et al., 2014; Gautam et al., 2015). Local gyrification index (LGI), the most common used scalar of cortical gyrification, is able to identify the changes of folding at each specific point of the cortical surface (Norbom et al., 2021). It has been implemented in studying cortical gyrification morphology primitively in neurodevelopmental disorders such as schizophrenia (Sasabayashi et al., 2020), autism spectrum and attention-deficit hyperactivity disorder (Gharehgazlou et al., 2021), affective disorders like major depressive disorder (Depping et al., 2018; Long et al., 2020) and bipolar disorder (Choi et al., 2022), extending to neuro-degenerative diseases like Alzheimer’s disease (Nunez et al., 2020). With a better understanding on the pathophysiology of PD that extends well beyond the nigrostriatal system, the cortical morphological changes of PD is gaining increasing interest in recent years. Studies have demonstrated alterations in cortical gyrification in patients with PD and suggested it as a potential metric for monitoring progression of PD (Zhang et al., 2014; Sterling et al., 2016; Li et al., 2020). Yet up to now the pattern of cortical gyrification in PD patients with depression has not been investigated.

Therefore, in this study, we used LGI to investigate the pattern of cortical gyrification among PD patients with and without depression, as well as the matched healthy controls, and evaluate the associations between LGI values from significant areas and depressive symptom scores. To further elucidate the effect of the severity of depression on cortical folding patterns in PD, we additionally analyzed the differences in LGI between ndPD and dPD subgroups divided with disease severity. The identification of specific cortical morphological differences between dPD and ndPD patients can shed new light on the neurobiological determinants of depression in PD, with clinical relevant implications for the diagnosis and management of PD-related depression.

## 2. Materials and methods

### 2.1. Participants

Participants involved in this research were recruited from the 2nd Xiangya Hospital, Central South University. All the PD patients were diagnosed according to the Movement Disorder Society Clinical Diagnostic Criteria for Parkinson’s disease (2015) (Postuma et al., 2015) by two experienced neurologists. Hoehn & Yahr (H-Y) scale and the motor part of the Unified Parkinson’s Disease Rating Scale (UPDRS-III) were used to evaluate the motor status of PD. Included patients were all with right handedness and age between 45 to 75 years old. To minimize the potential interference of motor symptoms in late disease stage and long disease duration, only patients with Hoehn & Yahr staging between 1 to 2.5 and disease duration no more than 5 years were included. Depression was diagnosed with the DSM-IV criteria by an experienced psychiatrist. The severity of depressive symptoms were assessed using 21-item Beck’s Depression Inventory (BDI): mild depression (10–16), moderate depression (17–29), severe depression (30–63) (Smarr and Keefer, 2011). Information on the use of anti-Parkinson drugs, including levodopa and pramipexole were collected. All participants underwent a semi-structured interview and were assessed with Clinical Dementia Rating scale (CDR) and the Mini Mental State Exam (MMSE). Participants with CDR scores>0.5 or MMSE scores lower than the corresponding education level were excluded (Li et al., 2016) to eliminate the interference of cognition impairment. Patients who had significant brain lesions, or had been ever diagnosed with depression or other neuropsychiatric disorders, or used antidepressants were also excluded. The Medical Research Ethical Committee of the 2nd Xiangya Hospital approved the study. Each participant provided written informed consent prior to study enrollment. All participants did not take any medicine 12 h prior to psychiatric assessment and MRI scanning.

We sought to recruit 30 non-depressed PD (ndPD), 30 mild-depressed PD (mi-dPD), 30 moderate-depressed PD (mo-dPD) and 30 severe-depressed PD (se-dPD) patients. With this sample size, with power of 0.8, and an alpha level of 0.05, we estimated to be able to detect effects at the level of effect size (ES) 0.65. Of the invited 120 early-stage PD patients, 9 were excluded due to potential cognitive impairment, 2 were excluded because of history of antidepressant medication, 8 due to incomplete clinical and questionnaire data, 5 due to unsatisfied imaging quality, 7 due to significant brain lesions in MRI (significant encephalomalacia, hydrocephalus, meningioma, acoustic schwannoma, large cyst), and 3 due to error during data preprocessing. Therefore, altogether 86 PD (27 ndPD, 24 mi-dPD, 21 mo-dPD and 14 se-dPD) patients and 43 healthy controls matched by age and sex were recruited.

### 2.2. Magnetic resonance imaging acquisition

All scans were conducted on a 3-Tesla Siemens scanner (Siemens Healthcare, Erlangen, Germany). Axial T2-weighted images were firstly obtained to exclude any dormant brain lesions. High-resolution 3D T1-weighted structural images was acquired using magnetization-prepared rapid gradient-echo sequence, with scan parameters as follows: repetition time = 1900 ms, echo time = 2.01 ms, slice number = 176, no gap, slice thickness = 1.0 mm, voxel size 1.0 × 1.0 × 1.0 mm, flip angle = 9°, field of view = 256 mm, matrix size = 256 × 256.

### 2.3. Preprocessing

The 3D T1-weighted images were preprocessed using FreeSurfer 5.3.0^1^ according to the standard auto-reconstruction algorithm. It mainly includes removal of nonbrain tissue, automated Talairach transformation, non-uniform intensity normalization, affine registration to the Montreal Neurological Institute space, and segmentation of gray/white matter tissue (Fischl and Dale, 2000; Park et al., 2021). The specific steps are as follows: First, import the raw MRI DICOM into FreeSurfer and verify the quality of the image (e.g., that the orientation is correct, the contrast sufficient, no scanner artifacts and head motion). Next, reconstruct the 3D cortical surfaces by creating two 3D mesh models composed of about 150,000 points for each hemisphere: a white (gray-white interface) and a pial (gray-CSF interface) surface. Then, the reconstructed data were visually inspected and any segmentation errors were manually corrected. Finally, compute the local Gyrification Index (lGI) for subsequent vertex-by-vertex group comparisons (Schaer et al., 2012). LGI is a three-dimensional extension of classical two-dimensional GI approach (Schaer et al., 2008). It measures the amount of the whole-brain cortical surface including those buried within the sulcal folds compared with the amount of visible cortical surface, which is neither restricted by sulcal walls nor biased by the orientation or thickness of the slices. Briefly, It is defined as the ratio between the area of the outer contour and the corresponding inner (pial) contour in an estimated spherical region of interest (ROI; 25 mm radius) based on a smooth surface constructed by FreeSurfer. Thus, the larger the LGI, the more complex the ditch and the higher the degree of cortical folding in the region.

### 2.4. Statistical analysis

Statistical Package for the Social Sciences (SPSS) version 25 was applied to perform Statistical analyses of demographic data and correlation analyses. One-way ANOVA was used to compare age, years of education, duration and MMSE, UPDRS III, BDI scores. Hoehn & Yahr and CDR were assessed using Kruskal–Wallis test. Gender, onset side of PD symptoms and the use of anti-Parkinson drugs among groups were analyzed using *χ*^2^ test. Relationships between average lGI values from significant regions and BDI scores within dPD patients were analyzed using Pearson’s correlation. *p* < 0.05 was considered significant.

To perform the vertex-by-vertex comparisons of the whole-brain LGI in Query Design Estimate Contrast (QDEC), the LGI values were projected on an average template for each subject and all of the individual reconstructed cortical surfaces were aligned to the template (Fischl et al., 1999a,b). Regional surface-based group differences in LGI at each vertex were analyzed using general linear model (GLM), with age and gender as covariates. A Gaussian kernel of 5-mm width was applied to smooth the LGI maps. The significant cluster was defined as *p* < 0.05, and Monte Carlo simulation was used for multiple-comparison correction. Average LGI values within significant clusters of the dPD patients were then extracted to perform correlation analysis with BDI scores. To further investigate the influence of the severity of depression on cortical folding patterns in PD, the differences in LGI between ndPD and dPD subgroups were additionally analyzed via QDEC with the same procedures.

## 3. Results

### 3.1. Subject characteristics

There were no significant group differences in age, gender, years of education, MMSE score, CDR score, and total intracranial volume among dPD, ndPD, and HC, as well as dPD subgroups (mi-dPD, mo-dPD, se-dPD). Specifically, the course and onset side of PD, H-Y classification, UPDRS III scores, and the distribution of the use of anti-Parkinson drugs between dPD and ndPD groups were also well-matched. As predicted, groups were significantly different in BDI scores with higher mean BDI scores in the dPD group (*p* < 0.001). Detailed demographics and clinical parameters of participants are summarized in Tables 1, 2.

**Table 1:** Demographic and clinical data of dPD, ndPD and HC groups.

	HC	ndPD	dPD	F/H value	*p* value
Patients, *n*	43	27	59		
Age	56.4 ± 7.1	57.2 ± 8.3	58.1 ± 8.3	0.616	0.542^a^
Gender (M/F)	22/21	15/12	32/27	0.153	0.926^b^
Education (yrs)	8.1 ± 3.3	7.2 ± 3.4	7.6 ± 3.0	0.752	0.474^a^
Onset side(L/R)	/	11/16	28/31	0.337	0.561^b^
Duration (yrs)	/	2.1 ± 1.4	2.4 ± 1.5	0.610	0.437^a^
H&Y, median (IQR)	/	1.5, (1.5)	1.5, (1.5)	0.063	0.802^c^
MMSE	27.2 ± 3.3	26.8 ± 3.2	26.4 ± 2.7	0.850	0.430^a^
CDR, median (IQR)	/	0.0 (0.0)	0.0 (0.0)	1.024	0.312^c^
UPDRS III	/	16.9 ± 8.6	20.1 ± 8.5	2.702	0.104^a^
BDI	4.5 ± 2.3	5.0 ± 2.9	21.0 ± 9.6	93.432	0.000^*^^a^
Anti-Parkinson treatment, *n* (%)					
Levodopa	/	12 (44.4)	32 (54.2)	0.711	0.399^b^
Dopamine receptor agonist (pramipexole)	/	4 (14.8)	10 (16.9)	0.062	0.803^b^
TIV (× 10^6^mm^3^)	1.42 ± 1.57	1.50 ± 1.24	1.47 ± 1.87	1.774	0.174

HC, healthy controls; ndPD, non-depressed PD patients; dPD, depressed PD patients; M, male; F, female; L, left; R, right; H&Y, Hoehn & Yahr stage; IQR, interquartile range; MMSE, Mini Mental State Examination; CDR, Clinical Dementia Rating scale; UPDRS III, the motor part of Unified Parkinson’s Disease Rating Scale; BDI, the 21-item Beck’s Depression Inventory; TIV, total intracranial volume. ^*^Indicates statistical difference between groups.

a One-way ANOVA.

b *χ*^2^test.

c Kruskal-Wallis test.

**Table 2:** Demographic and clinical data of dPD sub-groups.

	ndPD	dPD			F/H value	*p* value
		mi-dPD	mo-dPD	se-dPD		
Patients, *n*	27	24	21	14		
Age	57.2 ± 8.3	56.6 ± 8.4	59.2 ± 7.9	59.0 ± 8.7	0.515	0.673^a^
Gender (M/F)	15/12	13/11	12/9	7/7	0.186	0.980^b^
Education (yrs)	7.2 ± 3.4	8.5 ± 3.1	6.8 ± 3.0	7.2 ± 2.7	1.299	0.280^a^
Onset side(L/R)	11/16	12/12	10/11	6/8	0.520	0.915^b^
Duration (yrs)	2.1 ± 1.4	2.4 ± 1.5	2.1 ± 1.6	2.6 ± 1.6	0.581	0.629^a^
H&Y, median (IQR)	1.5, (1.5)	1.3 (1.4)	1.5(1.5)	2.0 (1.1)	3.362	0.339^c^
MMSE	26.8 ± 3.2	26.5 ± 2.8	26.4 ± 3.1	26.1 ± 2.2	0.198	0.898^a^
CDR, median (IQR)	0.0 (0.0)	0.0 (0.0)	0.0 (0.0)	0.0 (0.0)	2.951	0.399^c^
UPDRS III	16.9 ± 8.6	20.8 ± 7.1	20.0 ± 9.7	19.3 ± 9.3	0.978	0.407^a^
BDI	5.0 ± 2.9	12.9 ± 1.8	20.6 ± 3.5	35.5 ± 5.8	256.098	0.000^*^^a^
Anti-Parkinson treatment, *n* (%)						
Levodopa	12 (44.4)	12 (50.0)	10 (47.6)	10 (71.4)	2.907	0.406
Dopamine receptor agonist (pramipexole)	2 (7.4)	4 (16.7)	5 (23.8)	2 (14.3)	2.540	0.468
TIV (× 10^6^mm^3^)	1.50 ± 1.24	1.51 ± 1.90	1.42 ± 1.88	1.46 ± 1.77	1.152	0.333^a^

ndPD, non-depressed PD patients; dPD, depressed PD patients; mi-dPD, mild-depressed PD patients; mo-dPD, moderate-depressed PD patients; se-dPD, severe-depressed PD patients; M, male; F, female; L, left; R, right; H&Y, Hoehn & Yahr stage; IQR, interquartile range; MMSE, Mini Mental State Examination; CDR, Clinical Dementia Rating scale; UPDRS III, the motor part of Unified Parkinson’s Disease Rating Scale; BDI, the 21-item Beck’s Depression Inventory; TIV, total intracranial volume. ^*^Indicates statistical difference between groups.

a One-way ANOVA.

b *χ*^2^test.

c Kruskal-Wallis test.

### 3.2. Group comparisons of cortical gyrification

#### 3.2.1. ndPD patients versus HC

**Figure 1:**
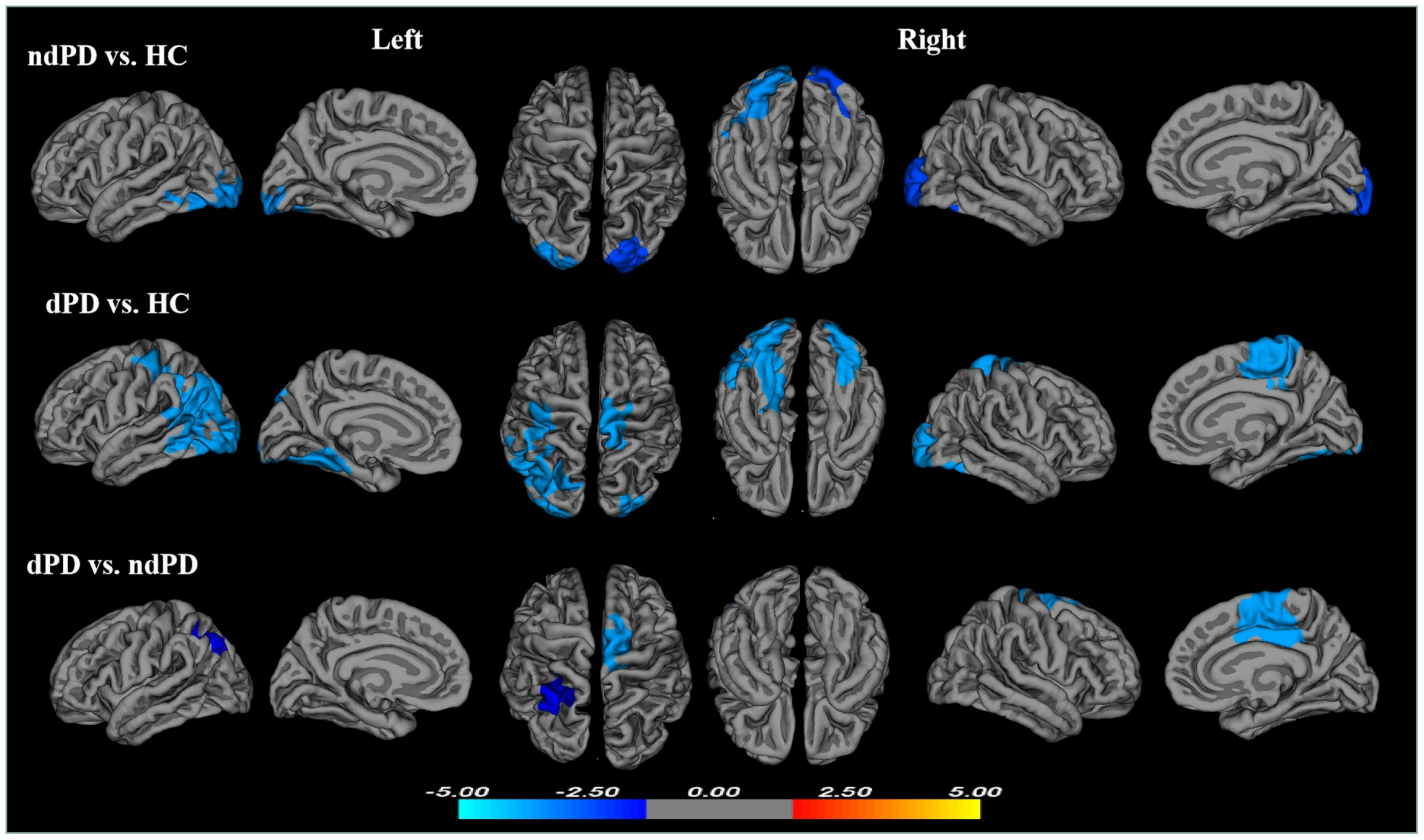
Clusters showing significant differences in gyrification of ndPD vs. HC, dPD vs. HC, and dPD vs. ndPD. Results of between-group analyses were thresholded at *p* < 0.05 with cluster-wise correction using Monte Carlo simulations. The color bar indicates *t*-values; blue color denotes hypogyrification (HC, healthy controls; ndPD, non-depressed PD patients; dPD, depressed PD patients).

Compared with HC, significantly reduced gyrification was observed in ndPD group across several regions of the cortex, including one cluster in the left hemisphere and one cluster in the right hemisphere. The left cluster had a peak vertex located within the fusiform gyrus covering portions of the occipital and inferior temporal gyri. The right cluster located on the lateral surface of the occipital lobe (Figure 1; Table 3).

**Table 3:** Brain regions showing significant group differences in gyrification.

Group comparison	Finding	Brain region	Cluster size (mm^2^)	Peak MNI coordinates			*p* value
				x	y	z	
ndPD Vs. HC							
	LGI ↓ in ndPD	Left fusiform gyrus, extending to lateral occipital and inferior temporal gyri	3,613	−34.2	−71.8	−9.0	0.00030
	LGI ↓ in ndPD	Right lateral occipital gyrus	3,083	30.4	−87.8	1.9	0.00460
dPD Vs. HC							
	LGI ↓ in dPD	Left superior parietal gyrus, extending to inferior parietal, post-central, middle temperal, inferior temperal, lateral occipital, fusiform and lingual gyri	14,058	−24.4	−73.6	25.3	0.00010
	LGI ↓ in dPD	Right paracentral gyrus	3,738	12.9	−35.8	61.9	0.00010
	LGI ↓ in dPD	Right fusiform, extending to lateral occipital gyri	2,942	34.5	−67.4	−7.9	0.00010
dPD Vs. ndPD							
	LGI ↓ in dPD	Left inferior parietal lobe, extending to superior parietal lobe	2037	−30.3	−62.5	41.0	0.04550
	LGI ↓ in dPD	Right posterior cingulate gyrus, extending to caudal anterior cingulate gyrus, superior frontal gyrus and paracentral lobule.	3,881	8.2	−6.7	37.7	0.00020
mo-dPD Vs. ndPD							
	LGI ↓ in mo-dPD	Right posterior cingulate gyrus, extending to superior frontal gyrus and paracentral lobule.	1,396	11.9	−7.5	38.5	0.00390
se-dPD Vs. ndPD							
	LGI ↓ in se-dPD	Left superior parietal lobe, extending to inferior parietal lobe	4,939	−24.9	−58.2	43.0	0.00010
	LGI ↓ in se-dPD	Left lingual gyrus, extending to parahippocampal, cingulate, fusiform gyri	2,493	−15.7	−41.2	−3.7	0.01290
	LGI ↓ in se-dPD	Right parahippocampal, extending to superior frontal, paracentral, cingulate, lingual, fusiform gyri	6,408	18.5	−35.3	−7.5	0.00010

HC, healthy controls; ndPD, non-depressed PD patients; dPD, depressed PD patients; mo-dPD, moderate-depressed PD patients; se-dPD, severe-depressed PD patients; LGI, local gyrification index; MNI, Montreal Neurologic Institute.

#### 3.2.2. dPD patients versus HC

Compared with HC, dPD group showed larger regions of reduced gyrification within three clusters. One cluster had a peak vertex located in the superior parietal gyri covering portions of the inferiorparietal, post-central, middle temperal, inferior temperal, lateral occipital, fusiform and lingual gyri in the left hemisphere. One cluster located in the right fusiform and lateral occipital gyri (peak localized in fusiform) and an additional cluster mapped onto the right paracentral gyrus (Figure 1; Table 3).

#### 3.2.3. dPD versus ndPD patients

**Figure 2:**
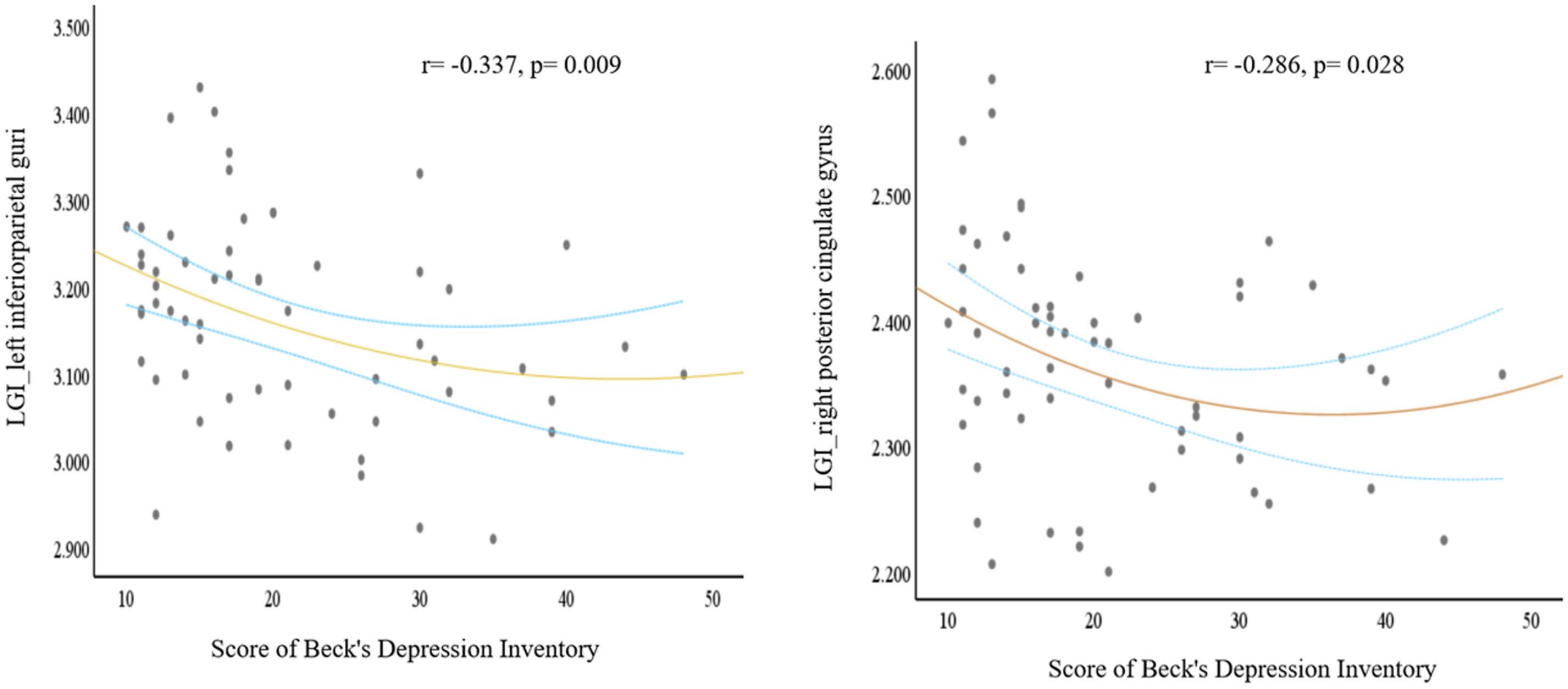
A plot for the signifcant correlation analysis. Based on the comparison between dPD and ndPD patients, local cortical gyrification (LGI) values of the significant clusters (one cluster peak localized in the left inferior parietal lobe, the other peak localized in the right posterior cingulate gyrus) negatively correlated with Beck’s Depression Inventory scores. The blue dashed curves depict the 95% confidence interval and the orange line shows the binomial regression fit. (dPD, depressed PD patients; ndPD, non-depressed PD patients).

The dPD group exhibited significantly reduced LGI relative to the ndPD group within two clusters. One cluster had a peak vertex mapped onto the left inferior parietal lobe comprising portions of the superior-parietal lobe, the other had a peak vertex located within the right posterior cingulate gyrus comprising portions of the right caudal anterior cingulate gyrus, superior-frontal gyrus and paracentral lobule (Figure 1; Table 3). The average LGI values in both the two clusters were negatively correlated with BDI scores within dPD patients (the left cluster: *r* = −0.337, *p* = 0.009; the right cluster: *r* = −0.286, *p* = 0.028, Figure 2).

**Figure 3:**
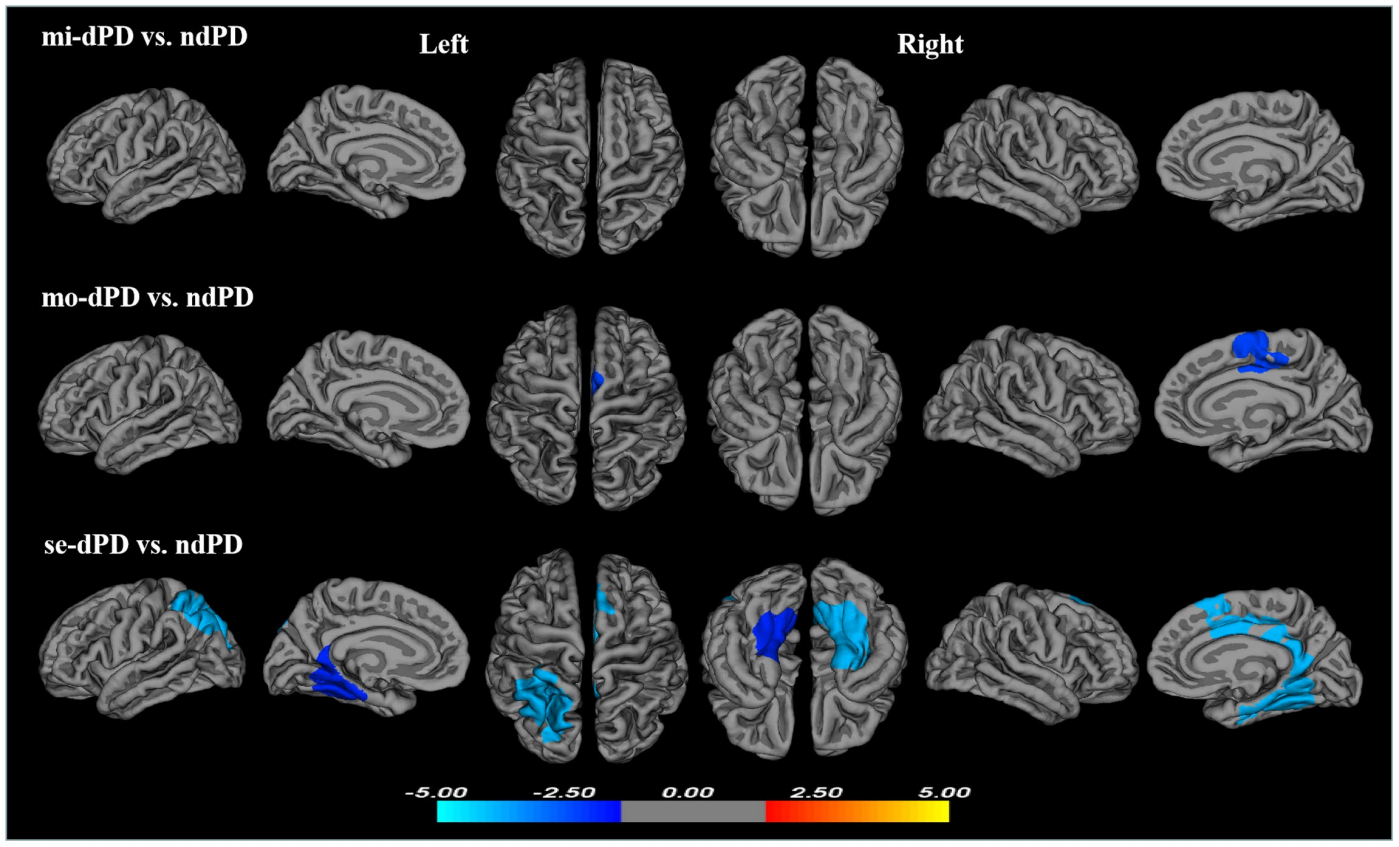
Clusters showing significant differences in gyrification of mi-dPD, mo-dPD, se-dPD, each compared to ndPD. Results of between-group analyses were thresholded at *p <* 0.05 with cluster-wise correction using Monte Carlo simulations. The color bar indicates *t*-values; blue color denotes hypogyrification. (ndPD, non-depressed PD patients; mi-dPD, mild-depressed PD patients; mo-dPD, moderate-depressed PD patients; se-dPD, severe-depressed PD patients).

To further elucidate the effect of the severity of depression on cortical folding patterns in PD, the comparisons between dPD subgroups and ndPD were conducted. Compared to ndPD, no significant differences of LGI was found in any brain areas in mi-dPD; mo-dPD showed reduced LGI in one cluster which had a peak vertex in the right posterior cingulate gyrus involving superior-frontal gyrus and paracentral lobule; while se-dPD exhibited more extensive reduction of LGI involving the left parietal, bilateral parahippocampal, cingulate gyrus, fusiform gyrus, lingual gyrus and the right superior-frontal, paracentral lobule (peak localized in the left parietal, the left lingual and the right parahippocampal region respectively, Figure 3; Table 3).

## 4. Discussion

In the present study, we investigated the differences in LGI between PD patients with- and without depression compared with matched controls, as well as between ndPD and dPD subgroups divided with disease severity. Three main findings emerged. Firstly, compared to ndPD, the dPD patients exhibited decreased LGI in the left parietal, the right superior-frontal, the posterior portion of cingulate and paracentral regions, and the LGI values within these areas negatively correlated with BDI scores within dPD group. Secondly, reduced gyrification was observed in mo-dPD and involving a larger region in se-dPD patients, but not in mi-dPD patients. Thirdly, both dPD and ndPD were characterized by a pattern of hypo-gyrifification in bilateral occipital regions involving potions of inferior temperal and fusiform cortex when compared to healthy controls. To our knowledge, this is the first study to explore the pattern of cortical gyrification in PD patients with- and without depression.

Although data on cortical gyrification in dPD patients remains lack, several possible explanations for these results could be speculated. In general, there are mainly two competing mechanical models for convolution formation during human brain development, i.e., differential growth theory (Richman et al., 1975) and axonal tension theory (Van Essen, 1997). The differential growth theory considered the differential growth rate between the outer and inner cortical layers as a major determinant of convolution formation (Richman et al., 1975). Among the few available cortical morphometric studies in dPD, abnormalities in cortical thickness (Luo et al., 2016; Chagas et al., 2017; Yin et al., 2022), density (Feldmann et al., 2008) and area (Huang et al., 2016) have been reported. These studies, though discrepant in results, revealed disorganization of the cortical architectures in specific regions which might account for the decreased cortical gyrifification observed in dPD.

While according to the tension-based theory of cortical morphogenesis, it is the mechanical tension along the axons in white matter that drives cortices fold in characteristic species-specific patterns (Van Essen, 1997). In other words, alterations in cortical gyrification may to some extent reflect changes of the white matter fibers or its connectivities. In dPD, white matter abnormalities have been frequently reported in previous diffusion tensor imaging (DTI) studies, which mainly involved the long contact fibers mainly connecting the prefrontal areas to the limbic or parieto-temporal regions, the cingulum and genu of corpus callosum (Huang et al., 2014; Ghazi Sherbaf et al., 2018; Wu et al., 2018; Ansari et al., 2019). Our previous study (Shen et al., 2022) has also revealed impaired white matter integrity in the left superior longitudinal fasciculus, anterior corona radiata, uncinate fasciculus, corticospinal tract, and bilateral inferior fronto-occipital fasciculus in severe-depressed PD when compared with ndPD patients. Moreover, fractional anisotropy values in the left superior longitudinal fasciculus negatively correlated with the BDI scores (Shen et al., 2022), well in accordance with the negative correlations between the LGI in the left parietal areas and BDI shown in the present study. In this regard, the reduced gyrification observed in this study seems more likely to be associated with the damaged microstructure or abnormal connectivity of the white matter that is adjacent to or spatially closely-connected to these regions.

Previous studies on patients with *de novo* depression have reported cortical gyrification abnormalities across multiple brain regions, including the left lingual gyrus, right posterior superior temporal sulcus (Long et al., 2020), precuneus, the superior parietal gyrus, the parahippocampal gyrus, the middle frontal gyrus and the fusiform gyrus (Depping et al., 2018; Chen et al., 2021). These areas, to not a small extent, differed from those of LGI reduction in dPD detected in the present study, which indicated characteristic pathophysiological mechanisms of depression in PD patients different from that in patients with *de novo* depression. Nevertheless, in the subsequent analyses between dPD subgroups and ndPD, while the mi-dPD showed no area with LGI reduction, the mo-dPD showed reduced LGI in the right posterior cingulate gyrus, superior-frontal gyrus and paracentral lobule. And the se-dPD exhibited more extensive reduction of LGI involving the parahippocampal, cingulate gyrus, fusiform gyrus, lingual gyrus, which largely overlapped with those reported in a previous VBM study exhibiting negative correlation between cortical volume and the severity of depression in dPD patients (Van Mierlo et al., 2015). These areas, mostly comprising part of the limbic system, have been constantly reported with reduced cortical gyrification in patients with *de novo* depression (Depping et al., 2018; Long et al., 2020; Chen et al., 2021). These results suggested that in the later stage of disease, depression in PD patients might partially share common neuropathological mechanisms with *de novo* depression (Prange et al., 2022). Future cortical morphometric studies including patients with primary geriatric depression are needed to further explore the neuropathological correlates of depression in PD patients and in general population.

Notably, mi-dPD patients showed no significant differences of LGI relative to ndPD, which, from the perspective of tension-based theory of cortical morphogenesis, was well in line with our previous DTI study detecting no white matter damage in the early stage of dPD (Shen et al., 2022). This result indicated that LGI might not be an optimal structural marker for the early detection of depression in PD patients. As depression aggravated, the mo-dPD and se-dPD exhibited extensive reduction of LGI, suggesting gyrification a potential marker for the progression of PD-related depression.

Another finding of this study was that compared with HC, both dPD and ndPD showed reduced gyrification in bilateral occipital regions covering potions of inferior temperal and fusiform cortex, which were rarely overlapped with those observed in dPD relative to ndPD. These results indicated that PD-related gyrification changes mainly involved the temporal-occipital regions, whereas depression in PD were associated with different patterns of gyrification change. Functional abnormalities and cortical morphological changes in temporal-occipital regions, which are known to be involved in high-order sensory processing, have been frequently reported in patients with PD (Luo et al., 2015; Wilson et al., 2019; Prajapati and Emerson, 2021; Tang et al., 2021). These alterations have been considered to be linked to impaired visual processing in these regions and visual-cognitive deficits in diagnosed PD (Weil et al., 2016). Moreover, the changes in cortical visual cortex might appear even before visual symptoms are clinically evident (Cardoso et al., 2010) and was considered as a potential marker of PD (Arrigo et al., 2017). Thus, our results showing reduced LGI in temporal-occipital regions in both dPD and ndPD are consistent with previous studies, supporting the involvement of the visual system in PD.

## 5. Limitations and future directions

Some limitations of this study should be taken into account when interpreting these results. First, more than a half PD patients in the present study had received anti-Parkinson medications. Although all participants did not take medicine at least 12 h prior to psychiatric assessment and MRI scanning, and the distribution of the use of anti-Parkinson drugs between dPD and ndPD groups showed no significant differences, It is hard to say the long-term influence of anti-Parkinson medications could completely be eliminated. Future studies with more detailed information on medications including their daily dose, frequency and how long they have been used, as well as with drug-naïve participants are warranted. Second, the sample size of this study, especially for the dPD subgroup analyses, was relatively small. Further work with a larger sample is needed to replicate our results. Third, as mentioned above, there was no subjects with primary depression. Future studies including age-matched subjects with primary depression may better locate the cortical gyrification alterations relating to depression in patients with PD. Last but not the least, although the effect of antidepressants have been reported to be associated with changes in different areas of the depression-related neural network in PD (Fregni et al., 2006; Morgan et al., 2018), whether the anatomical changes might be reversible with treatment of depression in PD (dPD) with antidepressants remains unclear. Future studies within a framework with longitudinal design, and incorporating both structural and functional imaging data are warranted to comprehensively investigate the neurobiological determinants of depression in PD, bringing out promotion on clinical management of PD-related depression.

## 6. Conclusion

In conclusion, the present study demonstrated that cortical gyrification is decreased within specific brain regions among PD patients with versus without depression, and those changes were associated with the severity of depression.

## Data availability statement

The raw data supporting the conclusions of this article will be made available by the authors, without undue reservation.

## Ethics statement

The studies involving humans were approved by The Medical Research Ethical Committee of the 2nd Xiangya Hospital. The studies were conducted in accordance with the local legislation and institutional requirements. The participants provided their written informed consent to participate in this study.

## Author contributions

CT contributed to the conception and design of the study. SC, QL, MW, CS, FZ, YL, JY, YT, and XL contributed to the data collection. QS contributed to the data analysis and writing the manuscript. HL contributed to the data collection and manuscript revision. JL contributed to the English language revision. All authors contributed to the article and approved the submitted version.

## Footnotes

^1^http://surfer.nmr.mgh.harvard.edu/
