# Corrigendum: Cortical gyrification pattern of depression in Parkinson's disease: a neuroimaging marker for disease severity?

**DOI:** 10.3389/fnagi.2024.1428166

**Published:** 2024-05-30

**Authors:** Qin Shen, Haiyan Liao, Sainan Cai, Qinru Liu, Min Wang, Chendie Song, Fan Zhou, Yujing Liu, Jiaying Yuan, Yuqing Tang, Xu Li, Jun Liu, Changlian Tan

**Affiliations:** Department of Radiology, The Second Xiangya Hospital, Central South University, Changsha, China

**Keywords:** Parkinson's disease, depression, severity of depression, magnetic resonance imaging, cortical gyrification

In the published article, there was an error in the Funding statement. The original Funding statement was as follows, “This study was supported by grants from Chinese National Science & Technology Pillar Program (grant nos. 2022YFC2009900/2022YFC2009904), the Natural Science Foundation of Hunan Province (grant no. 2022JJ30818 and 2021JJ40860), the science and technology innovation Program of Hunan Province (2021SK53502), and the Natural Science Foundation of Changsha City (grant no. kq2202416)”.

The correct Funding statement appears below.

